# CO and NO Coordinate Developmental Neuron Migration

**DOI:** 10.3390/ijms26167783

**Published:** 2025-08-12

**Authors:** Sabine Knipp, Arndt Rohwedder, Gerd Bicker

**Affiliations:** 1 Core Facility Imaging, Faculty of Medicine, Johannes Kepler University Linz, 4020 Linz, Austria; sabine.knipp@jku.at (S.K.); arndt.rohwedder@jku.at (A.R.); 2 Institute of Physiology and Cell Biology, University of Veterinary Medicine Hannover, Bischofsholer Damm 15/102, 30125 Hannover, Germany

**Keywords:** neural development, locust embryo, enteric nervous system, gaseous messenger signalling, chain migration, directionality

## Abstract

Similarly to the short-lived messenger nitric oxide (NO), the more stable carbon monoxide (CO) molecule can also activate soluble guanylyl cyclase (sGC) to increase cGMP levels. However, CO-induced cGMP production is much less efficient. Using an accessible invertebrate model, we dissect a potential interaction between the canonical NO/sGC/cGMP and CO signalling pathways during development. The embryonic midgut of locusts is innervated by neurons that migrate in four discrete chains on its outer surface. Transcellular diffusing NO stimulates enteric neuron migration via cGMP signalling. The application of an NO donor results in virtually all enteric neurons being cGMP-immunoreactive while CO increases cGMP production only in approximately 33% of the migrating neurons. Cellular CO release appears to act as a slow down signal for motility. We quantify how CO specifically increases the interneuronal distance during chain migration. Moreover, time-lapse microscopy shows that CO reduces the directionality of the migrating neurons. These findings support the function of NO and CO as antagonistic signals for the coordination of collective cell migration during the development of the enteric nervous system. These experiments and the resulting insights into basic scientific questions prove once more that locust embryos are not only preparations for basic research, but also relevant models for screening of drugs targeting NO and CO signalling pathways as well as for isolating compounds affecting neuronal motility in general.

## 1. Introduction

Cell migration is an essential feature of many morphogenetic events, such as gastrulation, blood vessel sprouting and nervous system development. The formation of the nervous system requires a directed migration of immature neuronal and glial cells from proliferative zones along specific pathways to the target locations of their somata. This dynamic feature is also characteristic for the enteric nervous system (ENS), in which enteric neuron precursor cells are generated in the neural crest before migrating towards and eventually populating the gut [1,2]. The insect enteric nervous system is composed out of linked peripheral ganglia and nerve plexuses that innervate the gut [3,4]. In contrast to vertebrates, the insect enteric nervous system covers the surface of the gut, providing ready access for visual observation and intervention in embryo culture [5]. Using mitotic activity and molecular cell surface markers, a comprehensive investigation performed by Ganfornina et al. mapped the development of the enteric ganglia, nerves, and nervous plexuses on the fore- and midgut of the locust *Schistocerca americana*’s embryos [6]. Post-mitotic midgut neurons, derived from three neurogenic zones on the foregut, assemble at the foregut–midgut boundary into four columns and rapidly move posteriorly in a directed chain migration. Eventually, the somata leave these migratory pathways and send terminal synaptic arborisations across the midgut musculature.

In a previous study, our lab explored the embryonic expression of the serotonergic transmitter phenotype in ENS ganglia and the midgut plexus of *Locusta migratoria*, a devastating agricultural pest insect that, like *Schistocerca americana*, can shift from a solitary to a gregarious phase and form giant swarms [7,8]. We observed that during chain migration on the midgut, ENS neurons leave trailing neurites behind which connect to the ingluvial ganglia. These neurites are fasciculated by expressing Fasciclin I [9], a cell-surface protein with a conserved domain found in a vertebrate and invertebrate protein family [10].

Based on small molecule inhibitor and rescue experiments in embryo culture, we identified the nitric oxide (NO)/cGMP, the cAMP/protein kinase A (PKA), and the carbon monoxide (CO) signal transduction pathways as regulators of neuronal migration on the midgut [9,11,12]. In the vertebrate brain, NO signalling is involved in cell proliferation, patterning, synaptogenesis, synaptic plasticity, and neurodegeneration [13,14,15,16,17,18,19,20]; similar functions of NO were also discovered in the insect brain [21,22,23,24,25,26,27].

In neurons, the free radical NO molecule is generated from L-arginine and oxygen by a Ca^2+^/Calmodulin-activated NO synthase (NOS) with nicotinamide adenine dinucleotide phosphate (NADPH) as a cofactor to facilitate this reaction. Therefore, a common technique for labelling NOS-expressing cells is mild formaldehyde tissue fixation followed by NADPH-diaphorase histochemistry (NADPHd) [28]. NO diffuses as a short-lived messenger from its production site across cell membranes and stimulates the heme sensor protein soluble guanylyl cyclase (sGC) to produce cGMP, an intracellular second messenger [20]. cGMP subsequently activates various downstream effector proteins, including cGMP-dependent protein kinases (PKG), phosphodiesterases (PDE), and cyclic nucleotide-gated ion channels [29]. Cell targets for nitric oxide can be identified by the capacity of NO to stimulate cGMP synthesis.

In well-defined regions of locust nervous tissue, measurements of Ca^2+^/Calmodulin-stimulated NOS activity in cell homogenates correspond to the neurochemical determination of NADPH diaphorase (NADPHd) activity and the resulting histochemical staining pattern of NADPHd-positive cells [30,31]. Using NADPHd staining as a histochemical marker for NOS, potential sources of NO have been identified in subsets of non-neural cells on the embryonic midgut [11]. After exposure to NO-releasing compounds, cellular cGMP production can be visualised with specific antisera [32]. MG neurons of the locust have been shown to express inducible cGMP-IR throughout the phase of migration and continue to show high levels of anti-cGMP staining in the lateral neurite branching phase and the formation of terminal processes [11]. When the midgut plexus acquires its mature configuration, cGMP-IR decreases (in [11]); therefore, NO-induced sGC activity in MG neurons is developmentally regulated and the timing of enzyme activity coincides with periods of neuronal motility as well as axonal outgrowth. Additional evidence for cGMP-mediated signalling came from experiments using a blocker of PKG. In embryos treated with a specific PKG inhibitor, MG neuron migration was retarded; this effect suggests that cGMP might influence migration via activating PKG.

Carbon monoxide is produced in neurons predominantly by the heme oxygenase 2 enzyme (HO-2) during the cleavage of heme as a reaction byproduct [14,33]. Neuronal stimulation can cause rapid activation of the HO-2 enzyme by phosphorylation through casein-kinase 2 [34]. Additionally, HO-2 can also be activated by binding of Ca^2+^/Calmodulin [35]. Since this constitutive HO isoform is Ca^2+^-regulated, CO is considered as an additional atypical messenger released in an activity-dependent fashion [14]. The catabolic breakdown of heme generates three compounds including CO, free iron, and biliverdin [36]. Since HO enzymes and CO production are embedded in an organismal network of immunological and anti-oxidative defence systems, many downstream signalling cascades remain possible [36,37,38]. Similarly to NO, CO has the potential to signal via the sGC/cGMP cascade [38,39].

In insects, heme oxygenase genes were described in *Drosophila* [40], *Apis* [41], *Anopheles* [42], and *Rhodnius* [43]. Using an antiserum against the constitutive isoform HO-2, we found that the enteric plexus neurons of *Locusta migratoria* express transient immunoreactivity while migrating on the midgut [9,12]. Blocking of HO enzyme activity in intact embryos by a metalloporphyrin inhibitor enhanced midgut neuron migration as opposed to the reduced migration which occurred after application of a CO donor. However, the detailed transduction pathways of the CO signal remain unresolved. Since both messengers can bind to sGC, but CO is less efficient than NO to stimulate cGMP formation [36], we suggested a competing mechanism as regulator for cGMP concentration and the migratory behaviour of the enteric neurons [12].

In this manuscript, we analyse the capabilities of NO and CO donors to raise cGMP-IR in enteric plexus neurons of *Locusta migratoria*. We re-examine how carbon monoxide regulates the interneuronal distance in the migrational chain and the maximum migrated distance of enteric neurons. We quantify the directionality of the migrating neurons through time-series microscopic observations of living tissue blots. Finally, we investigate the effects of blocking Rho-associated protein kinase (ROCK) with a small molecule inhibitor on the maximum migration distance. This gain of function provides further support for a NO/cGMP/PKG signalling pathway that controls via Rho/ROCK activity enteric neuron motility.

## 2. Results

### 2.1. Paracrine and Autocrine Gaseous Messengers Induce cGMP Production in Migrating Enteric Neurons

**Figure 1:**
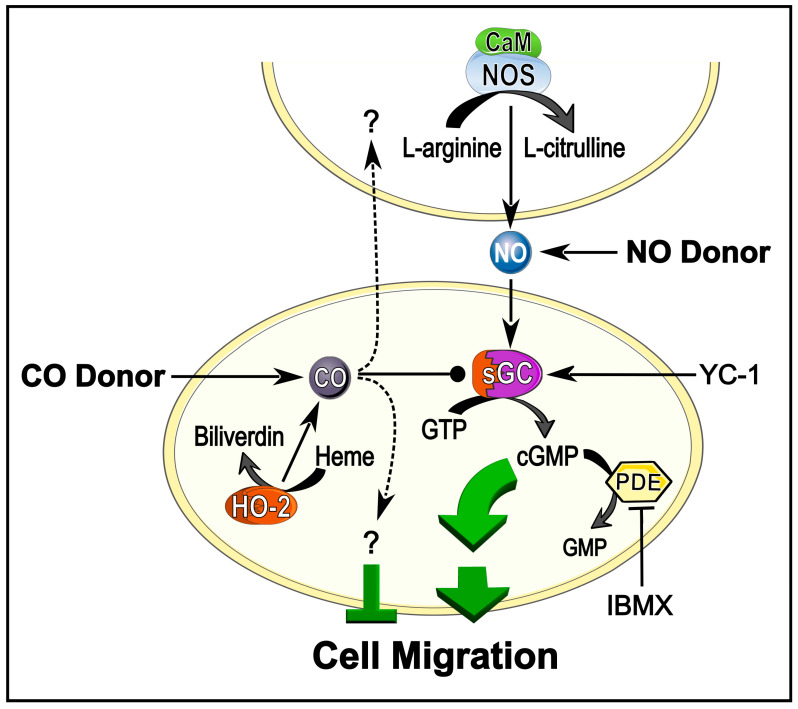
Carbon monoxide and nitric oxide/cGMP signalling cascades modulate cell migration. Schematic diagram illustrating the main steps of intra- and intercellular signalling and experimental manipulation of the pathways. Ca^2+^–Calmodulin (CaM)-activated nitric oxide synthase (NOS) releases nitric oxide (NO) during the conversion of L-arginine to L-citrulline. Diffusible NO binds to soluble guanylyl cyclase (sGC) in neighbouring cells and stimulates synthesis of cGMP from GTP. cGMP activates downstream signalling cascades to the cytoskeleton which result in enhanced cell migration. In parallel, intracellular heme oxygenase enzymes (HO), such as the constitutive isoform HO-2, release carbon monoxide (CO) as a byproduct during heme degradation to biliverdin. CO can bind to sGC and stimulate cGMP production, though with a rather modest increase in cGMP level compared to NO activation (indicated by blunt tip). Either NOS and HO activity can be imitated by application of NO and CO donors to the culture medium. Additionally, application of the sGC sensitiser YC-1 can increase cGMP production upon NO or CO binding. cGMP can be further enriched by inhibition of cGMP-degrading phosphodiesterases (PDE) via 3-isobutyl-1-methylxanthin (IBMX). (Diagram adapted from [12].)

**Figure 2:**
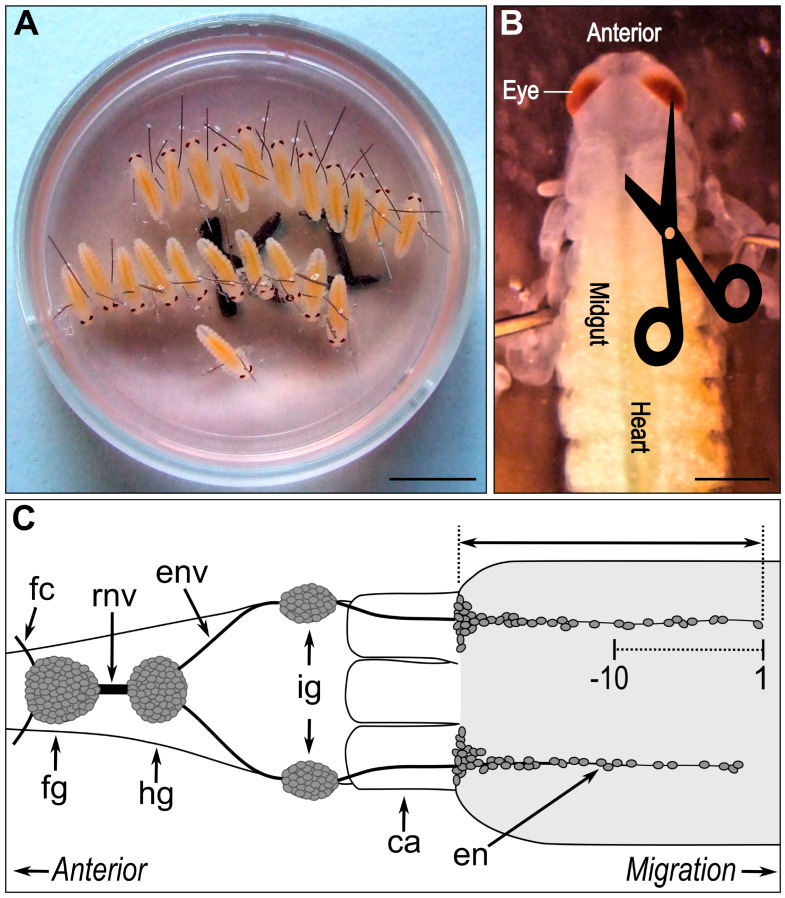
Graphical summary of the experimental setup to study enteric neuron migration in vivo. (**A**) Locusta embryos of the same clutch and developmental stage are immobilised in a Sylgard-embedded 35 mm Petri dish with L15 culture medium. (**B**) A small incision in the dorsal epidermis above the foregut allows access of culture medium and added pharmacological agents to the developing ENS. After 24 h of incubation, guts are dissected (visible here through the opaque epidermis as yellow, yolk-filled tube), enteric neurons are immunocytochemically labelled, and migratory tracks are analysed. Scale bars indicate 10 mm in (**A**) and 1 mm in (**B**). (**C**) Schematic drawing of the locust embryonic foregut and anterior midgut at 65% E in dorsal view (adapted from [12]) with midgut shaded in light grey and ganglia and neurons in dark grey. For quantification of the maximum enteric neuron migration, the distance from the foregut–midgut boundary to the leading enteric neuron was measured, as indicated by double headed arrow. Additionally, dispersion of enteric neurons along the migratory chain was calculated by measuring spreading of the leading ten neurons (indicated by scale below migratory pathway). ca, cecum; en, enteric neuron; env, oesophageal nerve; fc, frontal connective; fg, frontal ganglion; hg, hypocerebral ganglion; ig, ingluvial ganglion; rnv, recurrent nerve.

We have established a permissive, intercellularly transduced nitric oxide signal for migrating enteric neurons [11,12]. Using extracellular NO scavengers (hemoglobin) and NO synthase inhibitors (7-NI), enteric neuron migration was inhibited to the same extent for both compounds [12]. Contrary to this paracrine NO signal, enteric neurons can also produce CO as a second, more autocrine-acting messenger [9,12] (Figure 1). Manipulation of HO enzymes or adding CO donors pointed to a slowing down of migration by carbon monoxide [12]. With the help of refined in vivo embryo culture experiments (Figure 2), we were able to verify how enteric neuron migration is tuned by the NO and CO to the cGMP signalling cascade. This experimental setup facilitated an almost undisturbed development of the embryo. A small incision in the embryo neck (indicated by scissors in Figure 2B) enabled infiltration of any compound added to the cell culture medium, that was then distributed inside the embryo through its own heartbeat. To identify NO-responsive cells, we applied the widely studied NO donor sodium nitroprusside (SNP) after embryo culture and performed immunocytochemical labelling for cGMP, enabling the measurement of migrated distances of NO-sensitive neurons on the midgut (Figure 2C) [11,12].

**Figure 3:**
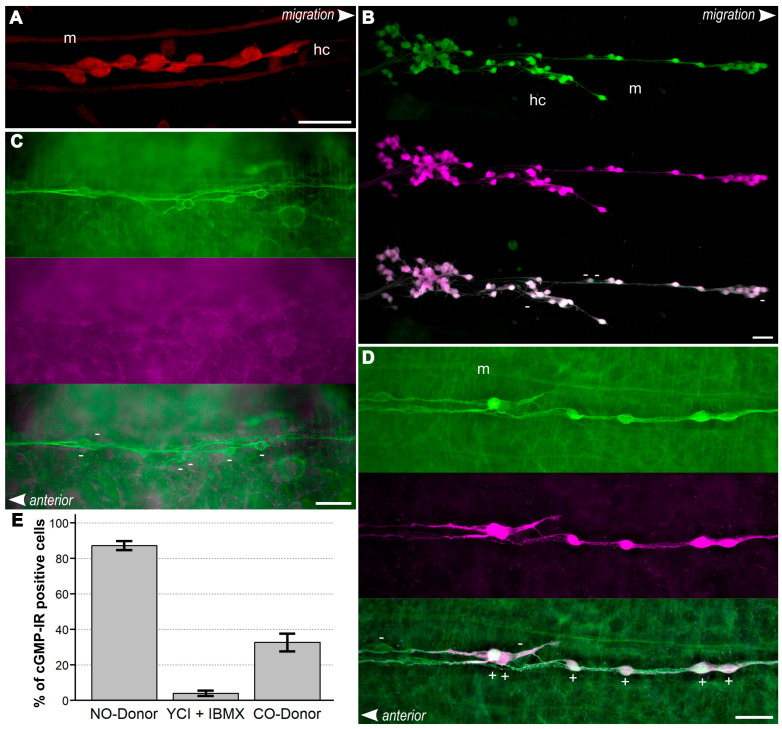
Immunocytochemical analysis of NO- and CO-dependent cGMP signalling in enteric midgut neurons. Fluorescence microscopy images of gut tissue blot preparations (**A**), whole mount locust embryo midguts (**C**,**D**), or confocal images of gut blot preparations (**B**) at stages between 65 and 75% of development (% E). Heme oxygenase (HO) immunoreactivity (IR) is depicted in red, cGMP-IR in magenta, horse radish peroxidase (**B**) or alpha-acetylated tubulin counterstaining (**C**,**D**) in green. Bottom panels are respective merged images. In merged images, (+) indicates cGMP-IR-positive and (−) cGMP-IR-negative cells, as judged and counted by microscopic observation. Anterior is to the left, cells are migrating to the right as indicated; scale bars represent 50 µm. (**A**) At 75% E virtually all enteric neurons express HO-2, as is true for the whole midgut migration [12]. (**B**) While virtually all neurons express high cGMP-IR after pre-incubation with an NO donor, almost no cGMP-IR can be detected after pre-incubation with sGC sensitiser YC1 and phosphodiesterase inhibitor IBMX alone (**C**). (**D**) Using a CO donor can elevate cGMP levels in migrating midgut neurons, though only in a third of the cells compared to NO activation. (**E**) Quantification of cGMP-IR-positive cells after pre-incubation as in (**B**–**D**). Midgut neurons were counted manually on independently prepared midgut samples. Sample numbers: NO donor = 5, YC1 + IBMX = 17; CO donor = 22. Depicted are mean percentages with SEM relative to total cell numbers derived from enteric neuron labelling. m, midgut musculature; hc, hemocyte.

Virtually all enteric neurons express heme oxygenase-2 immunoreactivity (IR) mainly in the somata during their midgut migration. Figure 3A depicts an exemplary microscopic image of HO-2-positive enteric midgut neurons in a gut tissue blot preparation. Neurons are clearly distinguishable from the background signal found in longitudinal midgut musculature (m) or hemocytes (hc). The enzyme is detectable in the neurons during their entire period of migration on the locust embryo midgut. This indicates the possibility of a constant intracellular release of CO in enteric neurons. Specificity of the HO-2 antiserum was verified in a previous study by immunocytochemical labelling of the ENS using pre-adsorption controls and an SDS-PAGE immunoblot analysis of protein composition from midgut lysates [12].

Similarly to nitric oxide, carbon monoxide is also able to stimulate cGMP production in enteric neurons (Figure 3B,D). For a rough quantification of the differential efficiency, cGMP-IR-positive cells (depicted in magenta in Figure 3) and total cell numbers on the midgut, as determined by neuronal marker labelling (green labelling in Figure 3), were manually counted and the ratio of cGMP-positive cells was calculated (Figure 3E). Immunofluorescence of alpha-tubulin or horse radish peroxidase (HRP) served as a cell type marker for insect neurons. The quantification results show up to 94% cGMP-IR-positive cells after pre-incubation with 100 µM nitric oxide donor (SNP, mean = 87%); however, only a third of cells (mean = 33%) show detectable cGMP-IR after stimulation with a CO donor (20 µM tricarbonyldichlororuthenium (II) dimer (CORM-II)). This is consistent with the reported approximate 30- to 100-fold-lower efficiency of the CO stimulation of sGC to convert GTP to cGMP [44,45]. The PDE inhibitor IBMX (1 mM) and sGC-sensitiser YC1 (25 µM) were used routinely in pre-incubation solutions for cGMP immunofluorescence labelling, but could not elevate cGMP enough to reach a meaningful IR detection level without a NO or CO donor present (Figure 3C,E).

### 2.2. Heme Oxygenase Activity Modulates Enteric Neuron Migration

**Figure 4:**
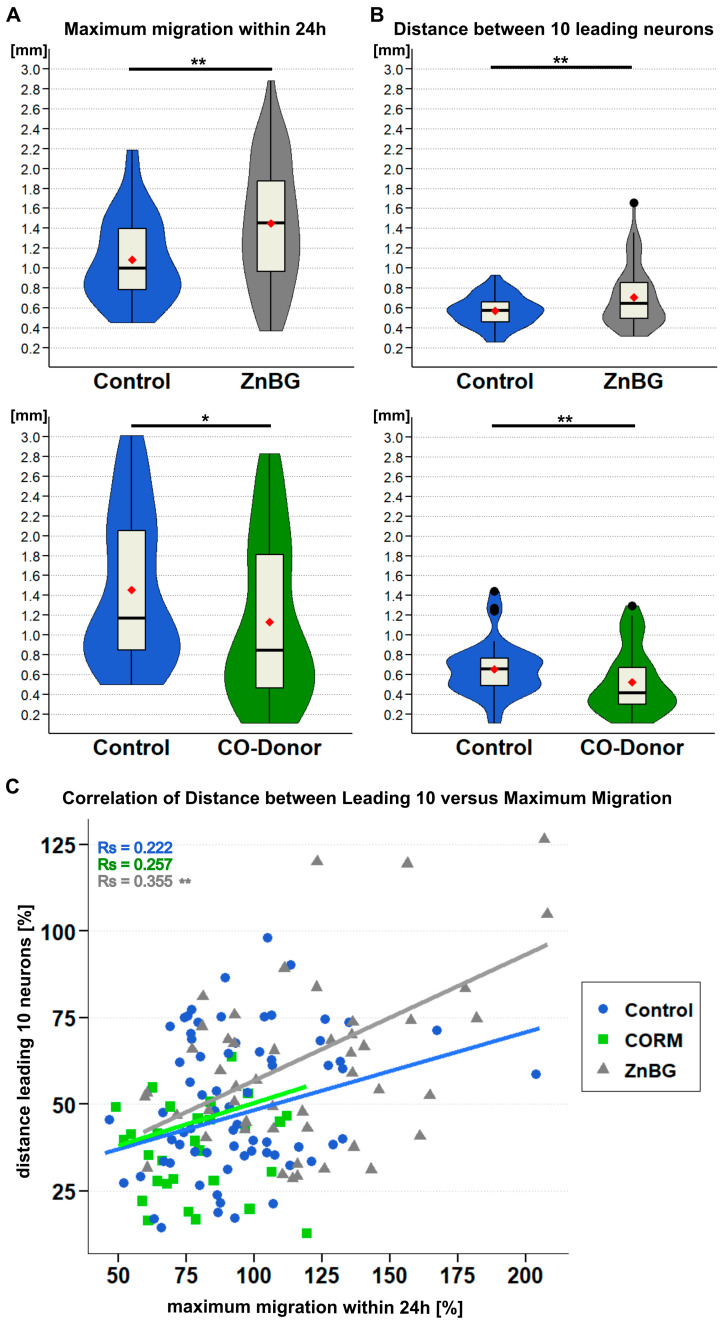
CO modulates interneuronal distance of migrating enteric neurons. Locust embryos were cultivated for 24 h as in vivo culture in L15 medium supplemented with DMSO (control group), 5 µM ZnBG, or the CO donor CORM-II (20 µM) diluted in DMSO. Data result from four independent experimental repeats, with total sample sizes of *n* = 40 for control and *n* = 55 for ZnBG, and three repeats with total sample sizes of *n* = 36 and *n* = 38 for CORM-II. Data for maximum migration (**A**) were previously gathered and originally published in [12]. Here we re-evaluated imaging data for additional analysis (**B**,**C**). (**A**,**B**) Data summarised in violin box plots with median values indicated by horizontal line; red diamond icons mark respective mean values, as in all following data plots. (**A**) Maximum migration distance covered by leading enteric neurons is significantly increased through inhibition of CO-releasing heme oxygenase enzymes (*p* = 0.00113), while application of CO donor reduces migration (*p* = 0.0198). (**B**) Leading ten enteric neurons of migratory tracks are significantly farther stretched if CO is decreased (*p* = 0.00473), but excess CO causes these neurons to accumulate (*p* = 0.00903). (**C**) Scatter plot illustrating relationship between maximum enteric neuron migration (**A**) and spreading of leading ten neurons (**B**) with respective regression lines for each condition. To allow for summary of all values from different experiment settings in one graph, individual values are given as percentage of respective experimental mean control (=100%). Spearman correlation coefficients are given in top left corner for control group in blue (*p* = 0.0626), CO donor in green (*p* = 0.1295), and ZnBG treatment in grey (*p* = 0.00812). *p* < 0.05 = *, *p* < 0.01 = **.

Lack of carbon monoxide due to inhibition of heme oxygenase enzymes with metalloporphyrins like zinc deuteroporphyrin 2,4-bis glycol (ZnBG, 5 µM) significantly enhanced midgut neuron migration from 1.082 mm (±0.069 mm; control) to 1.448 mm (±0.085 mm; ZnBG) (Figure 4A, displayed in grey). To further elucidate the nature of this gain of function, we re-evaluated and amended the previously produced experimental results. For each maximally migrated track per gut, we additionally measured the distance the respective leading ten enteric neurons covered. This revealed that a lack of CO significantly increased spreading of neurons along the migratory pathway (0.570 ± 0.024 versus 0.705 ± 0.040 mm, Figure 4B, top chart). Contrary to this increase in migrated distance, application of the CO donor CORM-II (20 µM) reduced the mean enteric neuron migration by almost 0.4 mm (1.515 mm ± 0.140 mm to 1.130 ± 0.131 mm). In line with this reduction in migrated maximum distance, excess CO also had an opposing effect on the distribution of the leading 10 enteric neurons (0.653 ± 0.046 mm versus 0.521 ± 0.055 mm, Figure 4A,B, displayed in green). This could certainly be expected, assuming that equal numbers of neurons have to stretch over a longer migratory distance or squeeze into shorter space. A correlation analysis of the data was performed (Figure 4C) to test a correlation between maximum track length and the dispersion of the respective leading 10 neurons; a significant positive correlation was confirmed in the case of HO enzyme inhibition (Spearman’s rank correlation Rho = 0.355, *p* = 0.00812; grey triangles in Figure 4C). No such correlation could be confirmed for application of a CO donor (Rho = 0.257, *p* = 0.1295, green squares) or control conditions (Rho = 0.222, *p* = 0.0626, blue dots). These different correlation values negate a simple expansion of cells due to there being a longer distance to travel, as a positive correlation should then be true for either experimental condition.

**Figure 5:**
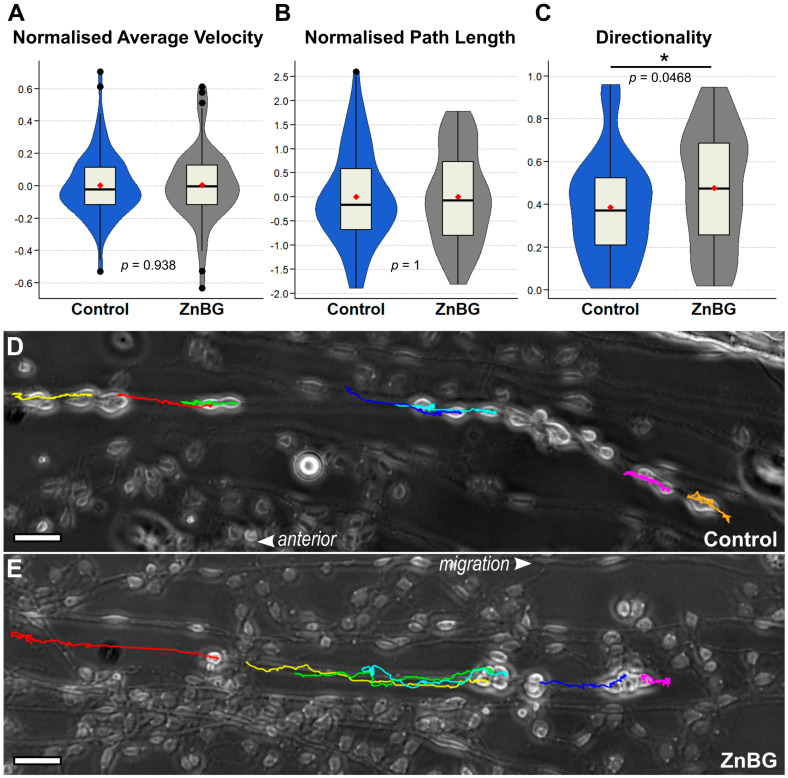
Live-cell-imaging-based analysis of enteric neuron migrational behaviour. Developing locust embryo ENSs were grown as gut tissue blots and microscopically imaged for up to 12 h, with 2-min image intervals. At least 10 individual enteric neurons were tracked and quantified for the first 8 h of the resulting image sequences (*n* = 95 from four experiments (control), *n* = 64 from five experiments (ZnBG)). Key variables are summarised as violin box plots. (**A**) Individual cell velocities were measured between frames and averaged over time. (**B**) Total track lengths were measured for individual cells. Raw data from (**A**,**B**) were normalised using z-scoring for each individual live cell experiment to reduce variations between experiments before summary and statistical analysis. (**C**) Directionality was calculated as the ratio between the shortest distance between individual cell start and end positions and the total path length accumulated by the same cell. (**D**,**E**) Exemplary endpoint images of time-lapse videos for control (**D**) and HO enzyme inhibition (**E**) after tracking of individual cells. Full videos can be found as Supplementary Videos S1 (= D) and S2 (= E). Scale bars represent 100 µm; the anterior is to the left, while cells migrate to the right. *p* < 0.05 = *.

We utilised live cell imaging to further investigate the enhanced migration due to HO enzyme inhibition. Blotted preparations of living embryonic MGs provided an easily accessible and flattened developing enteric nervous system with surrounding gut musculature and a functional migratory matrix [9,12]. Using such preparations from 63 to 65% of development (% E) embryos, we acquired image sequences from enteric neurons migrating along their already established migratory pathways. Key elements defining migratory behaviour were analysed in control conditions or with application of HO enzyme inhibitor ZnBG using ImageJ/FIJI plugins “MtrackJ” [46] and “Chemotaxis and Migration Tool” (ibidi GmbH, Gräfelfing, Germany). To cover a longer distance over the same time period (i.e., 24 h in vivo embryo culture), cells could be expected to migrate with a higher velocity. However, according to the gained live cell imaging data, average cell velocity did not change markedly when CO release was inhibited. Under either condition, cell speed corresponded roughly to a cell diameter per hour (Figure 5A, ca. 22 versus 23 µm/h). Likewise, total path length was not affected by HO enzyme inhibition (Figure 5B). We further checked for differences in the directionality, or efficiency, of cell migration, determined as the ratio between measured total path length (including all searching and potential migration errors) and shortest distance from start to end position [47]. Intriguingly, this directionality ratio differed significantly between control conditions (0.386) and HO-2 inhibition (0.476) (Figure 5C). With either treatment, one observed almost saltatory search-and-migrate behaviour in the leading cells (magenta and orange track in Figure 5D, magenta track in Figure 5E, and respective Supplementary Videos S1 and S2). However, follower cells exhibited differential behaviour depending on treatment. Cells in control medium supplemented with dimethyl sulfoxide (DMSO) seemed to be more explorative, sometimes even reversing their migration, thus accumulating total path length without gaining actual distance from their starting point. Enteric neurons in medium supplemented with ZnBG (diluted in DMSO) followed a rather straightforward directed migration.

### 2.3. Regulation of Enteric Neuron Migration Downstream of cGMP

**Figure 6:**
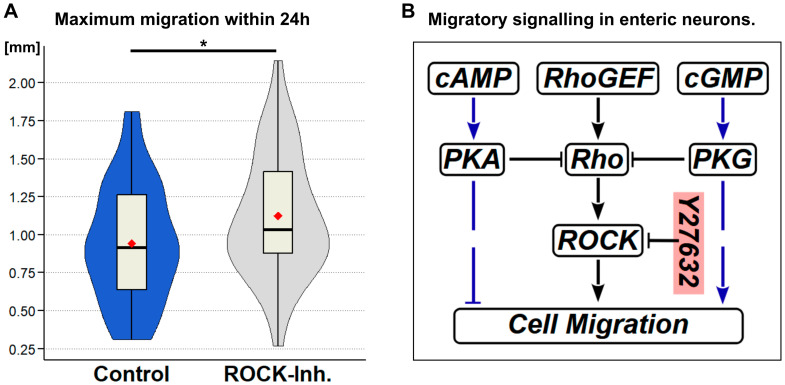
Signalling to the cytoskeleton downstream of cGMP. (**A**) Inhibition of Rho kinase with Y27632 significantly increases migration on the locust embryo midgut (*p* = 0.032). Maximum midgut migratory track lengths after 24 h in vivo culture with/without inhibition of Rho kinase with 100 µM Y27632 are summarised in violin box plots. Data result from four independent experiments, with total sample size of *n* = 42 for control group and *n* = 39 for ROCK inhibitor (ROCK-Inh.) treatment. (**B**) Summary of signalling pathways downstream of cyclic nucleotides. Opposing roles of cGMP/PKG and cAMP/PKA in locust embryo enteric neuron migration have been established in [11] (marked by blue pathway linker). Typical downstream targets of PKG are Rho GTPases. Rho GTPases regulate actin cytoskeleton dynamics via activation of Rho-associated protein kinase (ROCK) [50,51]. *p* < 0.05 = *.

Enteric neuron migration on the embryonic midgut requires NO/cGMP as a permissive signalling cascade and enlists cGMP-dependent protein kinase (PKG) as a major downstream effector [11]. A main target of PKG includes inhibition of Rho GTPases like RhoA [48,49]. Rho GTPases are known to regulate actin cytoskeleton dynamics and cell migration mainly via activation of Rho-associated protein kinase (ROCK) [50] (Figure 6).

To evaluate Rho/ROCK as a possible downstream target of cGMP/PKG in the locust embryo, we performed in vivo culture experiments just as for the NO/CO signalling pathway components. We used the cell-permeable and -selective ROCK inhibitor Y27632 at a concentration of 100 µM and analysed the resulting midgut neuron migration. Interestingly, ROCK inhibition generated an increase in maximum migration distance from 0.943 ± 0.060 mm to 1.132 ± 0.061 mm (Figure 6A). This correlated to a distance gain of approximately 24% for ROCK inhibition compared to a 30% increase after HO enzyme inhibition. This finding strongly indicates that enteric neuron migration in the locust embryo can be influenced by ROCK activity. However, from our findings, we are not able to exclude a cGMP-independent signalling cascade additionally regulating enteric neuronal migration via Rho/ROCK in locust embryos.

## 3. Discussion

In this publication, we focused on the contributions of NO and CO to the development of the enteric nervous plexus of the migratory locust. Similarly to other publications about the effects of NO signalling, we followed a common convention and measured the maximum distance [11,12,52] of the chain migration and determined the distance between the 10 leading neurons after intact embryo culture (Figure 4). We performed immunofluorescence labellings of enteric plexus (EP) neurons on whole mounts and tissue blots of the gut (Figure 3). In addition, we used time-lapse microscopic imaging to follow the tracks and obtain an estimate of the directionality of migrating neurons on the cultured tissue blots (Figure 5).

### 3.1. CO and NO Signalling and Embryonic Neuron Migration

The motivation to investigate CO-mediated signalling cascades comes often as a follow-up to research about the “celebrity” messenger NO, the molecule of the year in 1992 [53]. At physiological concentration, both messengers can signal via sGC-catalysed cGMP production [44,54]. Research spanning a couple of decades has contributed to the view that the two gaseous messenger molecules NO and CO are embedded in a network of other signalling cascades [14,37,38,54]. Apart from the canonical sGC/cGMP signal transduction pathway, the free radical molecule NO can also signal via redox mechanisms or s-nitrosylation of proteins [55,56,57,58]. Moreover, the synthesis of CO by HO also generates equimolar ferrous iron and biliverdin, the latter immediately reacting to bilirubin. Likewise, the many effects of CO cannot be uniquely attributed to sGC and cGMP, given its affinity to basically any heme protein [36,59]. Established downstream receptors for CO are, for example, mammalian epithelial Na^+^ channels, Ca^2+^- and voltage-activated K^+^ channels (BK channel), and cytochrome P450 oxidase systems [59,60,61]. Selective small molecules that target the biosynthetic enzymes NOS and HO and downstream pathways proved to be extremely useful tools to address the biological functions of the gaseous messengers NO and CO in the intact organism [12,62,63]. For immunofluorescent labelling, as in this publication, application of specific donor compounds raises the concentration of each messenger independently of physiological tissue levels (Figure 1). Enzymatic activity of sGC can be further sensitised for NO as well as CO activation by YC-1 and the resulting cGMP production can be accumulated by blocking its degradation to GMP with the PDE inhibitor IBMX [45]. This enables the sensitive immunocytochemical detection of cGMP according to the method of de Vente et al. [33].

The filopodia of the leading processes of all migrating neurons express NO-induced cGMP-IR [9,12]. Moreover, transcellular NO and NO-induced cGMP provide a permissive migratory signal for enteric neurons [11,12]. cGMP-IR was detected in more than 30% of the migrating neurons (including filopodia) following incubation with the CO-releasing compound CORM-II (Figure 3). Blocking of HO activity by ZnBG in the intact embryo led to a gain of function causing an increase in the maximum migrated distance (Figure 4A) and an increase in the interneuronal distance (Figure 4B,C). Further evidence that this effect is due to carbon monoxide comes from the observed loss of function with decreased migration after application of the CO donor CORM-II and the decrease in the interneuronal distance (Figure 4). The correlation of interneuronal distance versus whole track length illustrates how the distance between the migrating neuron gradually develops over the track length (Figure 4C). This phenomenon suggests that CO signalling might have an adaptive function in the coordination of the chain migration. Release of CO spreading from follower to leading neurons might signal them to slow down, promoting cellular advance at a similar speed and, thus, increasing cell cohesion.

Living gut tissue blots allowed for an overall posterior migration of enteric neurons and simultaneous microscopic visualisation, without movement artefacts, of the gut [9]. Enteric neurons adhered to a coated cover slip while surrounding muscle fibres and parts of the epithelial extracellular matrix were preserved. A former time-lapse imaging analysis of living gut tissue blots had already resolved the bipolar shape of the migrating neurons with leading processes and a trailing neurite connected to the ingluvial ganglia [9]. The highly motile chain of neurons migrated posteriorly between longitudinal muscle fibres, usually avoiding crossover of the muscle fibres. Individual cell movements experienced phases of relatively fast forward movements interspersed with phases of stopping and stationary filopodial searching behaviour [9]. Intriguingly, this mode of migration resembles the chain migration of neuronal precursors along the rostral migratory stream in rodents [64,65] which also express NO-sensitive sGC activity [66]. Our new video analysis indicates that under HO inhibition, neither the average velocity nor the total path length changes (Figure 5A,B), but the directionality of the migrating neurons is enhanced (Figure 5C). By sticking to a more direct path, neurons would inevitably cover more distance per time. Such a gain in directional migration could also amplify interneuronal distances (Figure 4B,C).

### 3.2. A Potential Interaction Between CO and NO Signalling

Based on immunochemical data and chemical manipulations of NO and CO signalling cascades, we proposed in our previous paper a scenario in which surrounding cells located in or on the gut provide a transcellular NO signal for stimulating sGC in the enteric midgut neurons (Figure 1) [12]. An additional CO pathway acting within or between the HO-positive midgut neurons provides an intra-/transcellular signal that downregulates motility. We suggested that the effects of CO on cell migration might arise out of a simple competition mechanism between CO and NO in regulating cGMP concentrations of the migrating neurons. While both molecules share the ability to activate sGC, CO activation is significantly less effective (about 4–5-fold) than NO (about 110–145-fold [44,45]. During release of NO and CO, part of the sGC molecules would bind CO, resulting in suboptimal cGMP production for motility (Figure 1) (cf. discussion in [12]). Our hypothesis was further supported through experiments involving the HO inhibitor ZnBG which resulted in a decrease in CO concentration. Thus, an increasing number of sGC molecules would be available for NO activation, stimulating the motility. Indeed, we could observe a gain of function for migration following HO enzyme inhibition. Other mechanisms acting directly at the conformation of the sGC enzyme have also been discussed [12]. A modulation of NO-derived sGC activity through endogenous CO resulting in decreased cGMP was shown, for example, in mammalian cerebellar granule cells or in rat retinas [39,67]. However, it is argued that, for instance, the about 1000× lower affinity of CO for sGC compared to NO makes such a simple competitive CO modulation of NO-sGC-cGMP signalling rather unlikely [38]. But then again, CO is also a much more stable molecule under physiological conditions, with a half-life of approximately 4 h compared to less than 2 s for NO [59], and in all given examples (including locust enteric nervous system), NOS is located in neighbouring cells, while HO is colocalised with sGC in the same cells. Nevertheless, to resolve these critical issues more data will be needed, e.g., from life cell cGMP imaging, that we currently do not have. Therefore, at this time, sGC/cGMP independent pathways cannot not be excluded.

Given that the experiments on the gut tissue blots reliably reflect the in vivo situation, the increased distance covered by the neurons under ZnBG influence (Figure 4A) would be explained by an enhanced directionality (Figure 5C). This interpretation of the newly derived data challenges a simple competition model in which NO and CO feed into a single signal transduction pathway, in which NO would enhance and CO impair motility via modulating cGMP levels (Figure 1). Additional unknown signalling pathways are necessary to explain how CO changes directed neuron migration from forward mode to more searching behaviour (Figure 5). These unknown signalling mechanisms may act in parallel to the sGC/cGMP pathway (indicated as “?” in Figure 1).

As outlined above, there is a multitude of possibilities as to how CO might influence the motility of the migrating neurons without modulating cGMP [14,36,38,61]. These possibilities range from cellular changes at the level of the plasma membrane to complex signalling cascades inducing cytoskeletal rearrangement. For example, CO generated by HO-2 requires oxygen. The outcome of this enzymatic reaction can directly influence the membrane potential in a physiologically meaningful way. In oxygen-sensing carotid body cells, the CO molecules released by the HO-2 enzyme serve to stimulate the opening of nearby large conductance Ca^2+^- and voltage-gated potassium (BK) channels [60]. The activation of BK channels leads to hyperpolarisation, leaving the membrane unexcitable. Potent hyperpolarising effects of CO on multiple ionic conductances have been found in uniquely identified Helisoma neurons [68]. Application of CO to these cultured neurons changes growth cone dynamics by increasing filopodial length. Here, a likely signalling pathway involves activation of sGC, PKG, and ryanodine receptors accompanied by low cytosolic Ca^2+^ levels [69].

To the best of our knowledge, we currently have no information on how and if any of the named examples affect cellular migration or whether CO and NO use cyclic nucleotide independent signalling cascades in locust embryo development. That said, our presented experimental data on modulation of enteric neuron migration via CO and sGC activation by either gaseous messengers could support an intracellular CO modulation of the transcellular NO signalling to sGC and thus modulate cGMP levels in migrating enteric neurons.

### 3.3. Signalling Pathways Downstream of Cyclic Nucleotide Formation

Cyclic GMP and small GTPases of the Rho family are central components for regulating the dynamic organisation of the actin cytoskeleton. Rho GTPases regulate actin cytoskeleton dynamics and cell migration, e.g., via activation of Rho-associated protein kinase (ROCK). Their activity induces stress fibre and adhesion formation and actin-myosin contractility, but also involves regulation of microtubule dynamics [50,51,70]. Rho/ROCK activity is also implicated in cell polarisation and directional cell migration [47,71,72] and can negatively regulate neuronal migration by contact inhibition [50]. Inhibition of sGC has been linked to alterations to the shape of immature neurons tangentially migrating to the cortex of the mammalian brain via the activation of the Rho signalling pathway [73]. Along these lines, we have also obtained evidence for cGMP-mediated signalling in intact embryonic locusts (Figure 6B) [11]. The sGC blocker ODQ retards midgut neuron migration. This effect can be rescued by application of cGMP [11]. Application of a specific PKG blocker (RPcGMPs) can also inhibit midgut neuron migration. These results suggest that cGMP might influence migration via activating PKG. Since the ROCK inhibitor Y27632 enhances maximum migration (Figure 6A), it is likely that a signalling cascade from cGMP via PKG and Rho will rearrange the actin cytoskeleton and influence cell migration. An increase in migration by Y27632 application was also shown for immature mammalian neurons in a Boyden chamber assay [73]. The authors proposed a signalling pathway from the NO-induced rise in cGMP via blocking of RhoA/ROCK activity to the promotion of neurite extension and increased migration. In locust embryos, rearrangement of F-actin between migrating and ODQ-induced stationary neurons could be visualised by fluorescence microscopy [11]. Similar experiments revealed that the cAMP pathway seems to play an inhibitory role in midgut neuron migration [11]. As shown in Figure 1 and Figure 6B, there are multiple opportunities for interactions of gaseous messenger and downstream cyclic nucleotide signalling pathways to coordinate enteric neuron migration.

### 3.4. Future Research Directions

Three issues have been identified as requiring future investigations. The first issue relates to the spatiotemporal dynamics of gaseous-messenger-mediated cellular processes. Successful attempts have been made to image with sufficient sensitivity the formation of NO in living vascular endothelial cells with a genetically encoded fluorescent indicator which detects NO to the nanomolar range [74]. To resolve how the NO/cGMP signalling pathway operates at physiological concentrations, a genetically encoded fluorescent cGMP biosensor has been expressed in cultured HEK cells [75]. Experiments with brief puffs of NO donor application resulted in a quantitative model for the optimal timing of NO stimulation to obtain effective cGMP formation [75]. The simultaneous real-time visualisation of calcium regulation and distinct NO generation of distinct NOS isoforms by genetic sensors have also been achieved [76]. Similar experiments with neural cells, and, in this context especially, insect neurons, remain to be explored. Because the role of CO as a physiological messenger has become increasingly accepted, there have also been attempts to monitor its concentration in living cells using genetically encoded sensors. One approach takes advantage of the unique CO-binding selectivity of CooA, a dimeric CO-binding heme protein from *Rhodospirillum rubrum* [77]. However, we are not aware of experiments using genetically encoded sensors for selective visualisation of CO levels in insect neurons. It remains a challenge to express the genetically encoded sensors within the migrating enteric neurons in a cell-specific manner.

The second issue relates to how results from basic research can be translated into tangible applications. Our basic research deals with a voracious pest insect and the development of its enteric nervous system, controlling the functioning of its feeding/digestive system [7,9,11,12]. A similar approach to prove a link between NO/cGMP signalling and midgut neuron migration in embryonic *Manduca* plexus obtained a different outcome [52]. In *Manduca* the migrating midgut neurons do also express NO-induced cGMP-IR. But in contrast to *Locusta*, inhibition of NO/cGMP signalling has no effect on the chain migration and there is no evidence for the presence of NOS near the migrating neurons [52]. However, EP development in *Manduca* differs slightly from *Locusta*, i.e., the somata of midgut neurons only migrate 20% along the midgut length before forming neurite branches and innervating the musculature [3,4]. Similarly to the development of the enteric plexus in *Manduca* [52], inhibitors of both NOS and sGC activity applied to locusts at later embryonic stages influenced neurite branch formation substantially [11]. During *Drosophila* embryo development, a migratory phase of neurons on the midgut is completely absent, subsequently leading to the lack of the midgut plexus [78,79]. Thus, development of the locust ENS is of special interest, as some locust neurons migrate along the entire length of the midgut (MG) towards the hindgut [6,11]. Despite there being common features of ENS formation in insects, these results show that there are distinctions and that the development in every species must be examined separately. We cannot expect to generate a selective insecticide simply by targeting a gaseous messenger signalling pathway during full completion of embryonic or larval development phases. On the other hand, species differences raise the possibility to target key developmental events specifically by a timed application of chemical agents, but this requires further investigation and analysis.

The final issue is the use of locust embryos for screening drugs that affect cell motility. Over the past two decades, we have found accumulating evidence for selective intervention in mechanisms of cell motility using the same small molecule ligands of NO and CO signalling that are also commonly applied on mammalian cells [7,9,11,12,80]. Developmental neurotoxicity (DNT) of chemicals poses a serious threat to human health worldwide. Based on findings that cellular mechanisms of growth cone guidance along molecular semaphorin gradients are conserved between embryonic locust and mammalian nervous systems, we have developed a predictive assay for testing the DNT potential of chemicals on axonal navigation of pioneer neurons in limb buds [81,82,83]. The advantage of this alternative in vitro testing assay is that intact locust embryos develop in serum-free culture together with the test chemicals. Again, the ROCK inhibitor Y27632 partially rescued axonal outgrowth inhibition after application of a pesticide. Since the next-generation calcium indicator dye Cal-520 AM can easily be bulk-loaded into locust neurons [84], it is possible to track cytosolic Ca^2+^ concentrations after testing chemical application. Based on research about the formation of the enteric plexus [6] and our investigations, a similar DNT and drug testing assay can now be initiated for the migrating cell somata of embryonic locust neurons.

## 4. Materials and Methods

Locust eggs (*Locusta migratoria*) were collected from our crowded animal culture, reared under standard conditions, and kept in moist Petri dishes at 30 °C prior to use. Embryos were staged by percentage of development (% E) according to Bentley et al. [85] with additional criteria for later embryos [86]. All chemicals were purchased from Sigma-Aldrich/Merck (Darmstadt, Germany) unless stated otherwise.

### 4.1. Anti-cGMP Pre-Incubation and General Immunocytochemistry

All steps of immunocytochemistry were performed at room temperature and with smooth agitation for whole mount preparations. For cGMP pre-incubation, embryonic whole mount gut or gut tissue blot preparations were collected in cooled Leibowitz 15 medium (L15, Gibco Life Technologies, Paisley, UK).

Pre-incubation for NO/CO-dependent cGMP immunocytochemistry on locust embryonic guts was carried out as already described [9,11,12,32]. In brief, freshly prepared specimens were incubated for 20 min at room temperature in L15 containing NO donor (sodium nitroprusside, SNP, 100 µM) or CO donor (tricarbonyldichlororuthenium (II) dimer (CORM-II), 20 µM), phosphodiesterase (PDE) inhibitor 3-isobutyl-1-methylxanthine (IBMX, 1 mM), and 3-(5-hydroxymethyl-2-furyl)-1-benzyl indazole (YC-1, 25 μM), a sensitiser of sGC. CORM-II, YC1, and IBMX were dissolved in dimethyl sulfoxide (DMSO), and SNP in L15.

All sample preparations were fixed in 4% paraformaldehyde (PFA) dissolved in phosphate-buffered saline (PBS, 10 mM sodium phosphate, 150 mM NaCl, pH 7.4) for 1–2 h at room temperature or overnight at 4°C. Preparations were permeabilised in 0.3% saponin in PBS for 30–60 min, rinsed in PBS containing 0.5% Triton X-100 (PBS-T), and blocked for at least one hour in 5% normal serum/PBS-T. Primary antibodies were diluted in blocking solution and incubated overnight at 4°C. Used primary antibodies and concentrations were as follows: sheep anti-cGMP (1:10,000–1:20,000), monoclonal mouse anti-acetylated α-tubulin (1:500–1:1000) and polyclonal rabbit anti-heme oxygenase 2 (1:400–1:1000, Stressgen, Victoria, BC, Canada), and polyclonal goat anti-HRP (1:5000, Jackson Immunoresearch, West Grove, PA, USA). After rinsing in PBS-T, guts were exposed for at least 2 h at room temperature or overnight at 4°C to biotinylated (Vector, Burlingame, CA, USA) or AlexaFluor 488-coupled (Molecular Probes, Eugene, OR, USA) secondary antibodies in blocking solution. Biotinylated secondary antibodies were visualised using fluorescent streptavidin-coupled dyes (Sigma-Aldrich/Merck, Darmstadt, Germany, Molecular Probes, Eugene, OR, USA). After washing in PBS-T and PBS, preparations were cleared in 50% glycerol (Roth, Karlsruhe, Germany)/PBS and mounted in 90% glycerol/PBS with 4% n-propyl-gallate. Control preparations incubated with 5% normal serum instead of primary antibodies and the subsequent detection system revealed absolutely no staining. For double labelling, ENSs were stained first for cGMP followed by acetylated α-tubulin or HRP immunocytochemistry.

### 4.2. In Vivo Culture Experiments

Locust embryo in vivo culture has been previously described in detail [9,11]. In brief, embryos of one clutch staged between 60 and 65% E were used. The optimal stage for start of in vivo culture is 63% E, a stage that can be easily determined by brownish pigmentation at the tips of the antennae. At this developmental time point, the gut and overlying epidermis have just closed and enteric neurons have started to invade the midgut. Eggs were sterilised in 70% ethanol, dissected in L15 medium, and randomly divided into treatment and control groups. Embryos were immobilised in Sylgard-embedded alcohol-sterilised Petri dishes and covered with cell culture medium supplemented with 1% penicillin–streptomycin. A small incision in the dorsal epidermis above the foregut allowed inflow of culture medium with small molecule inhibitors or the respective solvent to the developing ENS. See Figure 2A,B for a graphical summary. The used inhibitors were 5 µM zinc deuteroporphyrin-IX 2,4 bis glycol (ZnBG, Alexis, San Diego, CA, USA), 20 µM tricarbonyldichlororuthenium (II) dimer (CORM-II) pre-diluted in DMSO (resulting in less than 0.5% DMSO in culture medium), or the Rho-associated protein kinase (ROCK) inhibitor Y27632 (100 µM, diluted in L15). Following incubation for 24 h at 30 °C in the dark, whole guts were dissected and anti-cGMP and anti-acetylated α-tubulin double staining was performed. To analyse cell migration effects, we obtained fluorescence microscopic images of the labelled specimen and measured the distance from the foregut–midgut boundary to the position of the leading enteric neuron (Figure 2C, indicated by double-headed arrow) using ImageJ/FIJI [87,88]. Only the maximum value for migration on each midgut was included in the respective sample groups. We further measured the distance between the first to tenth migrating enteric neurons for each of the maximum migratory tracks (as indicated by the scale below the top migratory track in Figure 2C) to determine information on the dispersal of individual cells along the pathway.

### 4.3. Gut Tissue Blot Preparation and Life Cell Imaging

Alternatively to dissected whole mount guts, we also generated “gut tissue blot” preparations. This type of preparation eliminates the background caused by yolk inside the whole mount guts and results in a flat in situ enteric nervous system suitable for confocal and life cell microscopy [9,12]. In short, dissected guts were rolled carefully over a poly-D-Lysin coated cover slip (dilution 1:100) in a Petri dish filled with L15 culture medium. During this procedure, enteric nervous systems including neurons of the forming plexus and ganglia as well as muscle fibres and haemocytes adhered to the coated surface, whereas the intact epithelium and yolk could be removed.

Tissue blot preparations were allowed to settle for 30 min at room temperature to ensure sufficient adherence prior to further treatment, i.e., fixation and immunocytochemistry (see above) or life cell imaging. For life cell imaging we used tissue blots generated from embryos staged 63–65% E. Settled tissue blot preparations were washed with sterile L15 supplemented with 1% penicillin–streptomycin solution (L15-PS) and incubated for at least 1 h at 30 °C before time-lapse imaging in a PTC-10npi warming heater (30 °C) on the stage of a Zeiss Axiovert 200 microscope equipped with a Photometrics Cool Snap digital camera and associated MetaFluor Imaging software. Phase contrast images were captured at 2 min intervals for a minimum of 12 h. AVI-Videos were created using ImageJ/FIJI and image quality was carefully enhanced by adjusting brightness and contrast if necessary. Single cells were tracked using the “MTrackJ” plugin [44] and key migratory features like velocity, path length, and directionality were determined with the same plugin and the “Chemotaxis and Migration Tool” from Ibidi (as ImageJ plugin, developed by Gerhard Trapp and Elias Horn, ibidi GmbH, Gräfelfing, Germany). Only data from the first 8 h of the image sequence were used for quantifications. Individually identifiable cells from all parts of the migratory chain were traced in a blinded analysis. To reduce variations between individual time-lapse imaging experiments, a z-score normalisation was carried out in RStudio for each sequence for average velocity and path length. This was assumed necessary, e.g., as live cell imaging was performed without a dedicated climate chamber or microscope room, thus, specimens were exposed to changing environmental conditions (air pressure, light, humidity, etc.). Directionality was calculated as the ratio between the shortest distance between the cell position at start of imaging sequence and its end position after 8 h and the total migrated path length for the same individual cell [47].

### 4.4. Microscopic Image Acquisition and Processing

Phase contrast images were acquired on a Zeiss Axiovert 200 microscope equipped with a Photometrics Cool Snap digital camera and associated MetaFluor Imaging software (Teledyne, Birmingham, UK). Fluorescence images were acquired using a Zeiss Axioscope microscope equipped with an Axiocam3900 digital camera linked to a Zeiss image acquisition system (Zeiss Axiovision, Carl Zeiss, Jena, Germany). Confocal images of selected preparations were taken with a Leica TCS SP2 or SP5 confocal microscope using Leica LCS or LasX software (Leica Mikrosysteme, Wetzlar, Germany). If necessary, images were processed with ImageJ/FIJI (Version 1.54 and earlier) for adjustment of brightness and contrast and colour conversion. Figure panels were arranged using inkscape (inkscape.org, version 0.92) and GIMP (GNU image manipulation program, gimp.org, version 2.10). Parts of Figure 1 were drawn using images adapted from Servier Medical Art (https://smart.servier.com/, accessed on 15 March 2025), licenced under CC BY 4.0 (https://creativecommons.org/licenses/by/4.0/, accessed on 15 March 2025).

### 4.5. Statistical Analysis and Data Visualisation

The open-source R/RStudio IDE software (version 2024.09.1, Posit Software, Boston, MA, USA) was used for all statistical analyses and visualisation of acquired data. Used scripts are available on reasonable request.

For data summary, normalisation, and visualisation, RStudio package *tidyverse* [89] was used. For statistical data analysis, the *rstatix* package [90] was employed. Normal distribution of samples was tested using the Shapiro–Wilk test with *p* > 0.05 rated as normal sample distribution. The Shapiro–Wilk test is deemed most suitable for smaller sample sizes, rather than, e.g., Kruskal–Wallis [91]. For normally distributed samples, Welch’s *t*-test was applied for statistical comparison, while non-normal distributed samples were compared with a two-sided Wilcoxon signed rank test. Spearman correlation analyses (Figure 4) were performed using the basic R/RStudio *cor.test* function and *cocor* package for statistical comparison of correlations (n.s.) [92]. Statistical significance was considered for *p* < 0.05 = *, *p* < 0.01 = **, *p* < 0.001 = ***.

## Data Availability

The data supporting the conclusions presented in this paper, or RStudio scripts for data analysis, can be made available by the authors on reasonable request after publication.

## Supplementary Materials

The following supporting information can be downloaded at: https://www.mdpi.com/article/10.3390/ijms26167783/s1.
