# Supplementary material for: CO and NO Coordinate Developmental Neuron Migration
Source: Int J Mol Sci. 2025 Aug 12;26(16):7783. doi: 10.3390/ijms26167783 (PMC12387027; doi:10.3390/ijms26167783)
Supplement: Supplementary file 1 [file ijms-26-07783-s001.zip › Supplementary Videos/SupplVideoLegends.pdf]

CO and NO coordinate developmental neuron migration

Sabine Knipp, Arndt Rohwedder and Gerd Bicker

Supplementary Material - Legends

Video S 1. *Time laps microscopy of enteric neuron migration in control conditions.*

Exemplary time lapse video with tracking of enteric neurons for control conditions (L15 + DMSO. Developing locust embryo ENSs were grown as gut tissue blots and imaged for 12h. Images interval was two minutes, to reduce file size only every second image is used for the video montage. Anterior is to the left. Scalebar represents 100 µm.

Video S 2. *Time laps microscopy of enteric neuron migration with HO enzyme inhibtion.*

Exemplary time lapse video with tracking of enteric neurons with ZnBG added to the cell culture medium L15. Developing locust embryo ENSs were grown as gut tissue blots and imaged for 12h. Images interval was two minutes, to reduce file size only every second image is used for the video montage. Anterior is to the left. Scalebar represents 100 µm.
